# Unilateral drainage and chemotherapy prolong the patency of a plastic stent placed above the sphincter of Oddi in patients with malignant hilar biliary obstruction

**DOI:** 10.1002/deo2.404

**Published:** 2024-07-15

**Authors:** Fumimasa Tomooka, Koh Kitagawa, Akira Mitoro, Yukihisa Fujinaga, Norihisa Nishimura, Tadashi Namisaki, Takemi Akahane, Kosuke Kaji, Shohei Asada, Shinya Sato, Jun‐Ichi Hanatani, Hitoshi Mori, Yuki Motokawa, Tomihiro Iwata, Hiroki Kachi, Yui Osaki, Hitoshi Yoshiji

**Affiliations:** ^1^ Department of Gastroenterology Nara Medical University Nara Japan; ^2^ Division of Endoscopy Nara Medical University Nara Japan

**Keywords:** chemotherapy, endoscopic retrograde cholangiopancreatography, inside stent, malignant hilar biliary obstruction, unilateral drainage

## Abstract

**Objectives:**

To evaluate the results of inside stent therapy for unresectable malignant hilar biliary obstruction and identify factors related to stent patency duration.

**Methods:**

Of 44 patients who underwent initial inside‐stent placement above the sphincter of Oddi from April 2017 to December 2022, 42 with the resolution of jaundice (clinical success rate, 95.5%) were retrospectively analyzed. Univariate and multivariate logistic regression analysis identified factors associated with stent patency duration.

**Results:**

Univariate analysis revealed significant differences in the drainage method (406 days for unilateral drainage vs. 305 days for bilateral drainage of the right and left liver lobes, *p* = 0.022) with or without chemotherapy (406 days with vs. 154 days without, *p* = 0.038). Multivariate analysis (Cox proportional hazards analysis) revealed similar results, with unilateral drainage (*p* = 0.031) and chemotherapy (*p* = 0.048) identified as independent factors associated with prolonged stent patency. Early adverse events were observed in two patients (4.8%; one cholangitis, one pancreatitis).

**Conclusions:**

Inside‐stent therapy was safely performed in patients with malignant hilar biliary obstruction. Simple unilateral drainage and chemotherapy may prolong stent patency.

## INTRODUCTION

Endoscopic biliary drainage is a well‐established palliative treatment option in patients with malignant hilar biliary obstruction (MHBO).^1^, ^2^, ^3^ Conversely, the choice of either a plastic or metal stent for biliary drainage in patients with MHBO or the choice of unilateral or bilateral drainage in the right or left lobes of the liver remains controversial.^1^, ^2^, ^3^ In addition, recent advances in chemotherapy for pancreatic cancer, biliary tract cancer, and hepatocellular carcinoma are remarkable.^4^, ^5^, ^6^ When self‐expandable metallic stents (SEMSs) are used for biliary drainage in patients with MHBO, they are expected to prolong patency longer than plastic stents (PSs); however, reintervention is often technically difficult.^7^ Conversely, PSs can be easily removed, and replacement is technically easier than that with SEMSs. Therefore, PSs are increasingly used in palliative biliary drainage in patients with unresectable MHBO.^8^, ^9^, ^10^ However, the most serious issue of conventional PSs is their short patency.

On the contrary, the usefulness of “inside‐stent” therapy, wherein a threaded PS is placed above the sphincter of Oddi, has been reported.^11^ It has been suggested that stent patency might be longer for an inside stent than for a conventional PS because it can block duodenal juice reflux that contains bacteria. However, to the best of our knowledge, no studies have examined the factors that affect the stent patency of inside stents. This study aimed to conduct a retrospective analysis of the results of inside‐stent therapy for unresectable MHBO and identify factors related to stent patency duration in 42 patients.

## METHODS

### Study population

Overall, 44 patients with consecutive unresectable MHBO underwent inside‐stent treatment between April 2017 and October 2022 in our hospital. The MHBO diagnosis was based on the pathological and radiological findings or clinical courses. Two patients who received inside‐stent therapy via endoscopic retrograde cholangiopancreatography (ERCP) but whose jaundice did not improve were excluded from the study (clinical success rate; 95.5%).

This study was approved by the Institutional Review Board of Nara Medical University Hospital (IRB No: 2701) and performed according to the Helsinki Declaration of the World Medical Association. Written informed consent was obtained from all patients before performing ERCP. An opt‐out option on the website was used instead of written informed consent for inclusion in this study owing to the retrospective nature of this study.

### Endoscopic procedures and stent placement

**FIGURE 1:**
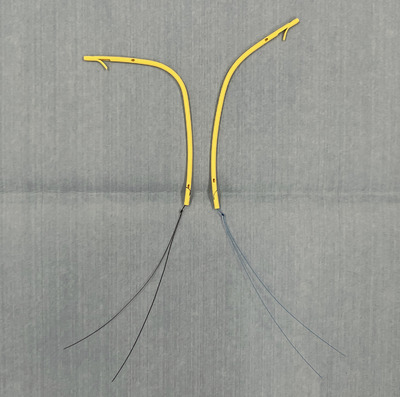
Plastic “inside‐stent” with thread. Two different proximal‐end configurations of stents are available to accommodate the bile‐duct bends. Flaps are equipped only on the proximal end of the stents.

ERCP was performed with the patients in a prone or semi‐prone position under conscious sedation using intravenous midazolam and buprenorphine hydrochloride or dexmedetomidine with CO_2_ insufflation. A standard therapeutic duodenoscope (JF260V/TJF260V; Olympus Optical) was used to perform all the procedures. Following selective bile‐duct cannulation, cholangiography was performed to examine bile‐duct stenosis. A threaded biliary plastic stent (Through & Pass; Gadelius Medical Co. Ltd., Figure 1) was placed above the sphincter of Oddi for MHBO during ERCP. The decision to perform unilateral or bilateral drainage was based on preoperative imaging studies to achieve at least 50% liver volume. However, regardless of liver volume, stenting was not performed in hepatic regions where portal blood flow was obstructed by tumor invasion. These evaluations were performed using contrast‐enhanced computed tomography and magnetic resonance imaging study. Following successful stent placement, the decision to replace it periodically or after recurrent biliary obstruction (RBO) was made by the physician in each case.

### Endpoints and definitions

The primary study endpoint was to determine the factors affecting stent patency in inside‐stent therapy for patients with MHBO. The secondary endpoints were the success rate of the endoscopic procedure and the incidence of adverse events (AEs) associated with the endoscopic procedure. All terms used in this study were those in the TOKYO criteria 2014 for transpapillary biliary stenting.^12^ The RBO time point was defined as the point at which clinical features related to the obstruction or migration of the inside stent were observed. Time to RBO (TRBO) was defined as the period between the first inside‐stent placement and RBO. For patients who underwent periodic stent replacement before RBO occurrence, the replacement date was defined as censored. For patients who could not be followed up because of transfer to another hospital, the date of last confirmed patency was defined as censored. For patients who died before RBO occurrence, the date of death was defined as censored. The clinical success was defined as a decrease in the serum total bilirubin level of <2.0 mg/dL or a 50% decrease within 14 days after treatment relative to the pretreatment level. The American Society of Gastrointestinal Endoscopy dictionary was used to grade AEs.^13^ The causes of stent dysfunction were determined according to the TOKYO criteria 2014 for transpapillary biliary stenting.^12^ We analyzed the following factors to evaluate their effects on stent patency: preoperative cholangitis, Bismuth classification (I vs. II–VI),^14^ ES, stent placement method (unilateral vs. bilateral), chemotherapy treatment after inside‐stent placement, endoscopic nasobiliary drainage (ENBD) management before inside‐stent placement, and primary disease (biliary vs. nonbiliary tract cancer).

### Statistical analysis

Chi‐square test or Fisher's exact test was performed for comparing the categorical variables, and the *t*‐test, or Mann–Whitney *U*‐test was performed for comparing continuous variables. TRBO and survival were subjected to Kaplan–Meier analysis and the curves were compared via the log‐rank test. The associations between stent patency and seven parameters were evaluated via univariate and multivariate Cox proportional hazard model analyses. Statistical significance was *p* <0.05.

EZR ver. 1.41 (Saitama Medical Center, Jichi Medical University) supported by R ver. 4.0.3 (The R Foundation for Statistical Computing, Vienna, Austria) GUI, EZR ver. 1.41 (Saitama Medical Center)^15^ was used to perform all statistical analyses.

## RESULTS

### Patient survival

**FIGURE 2:**
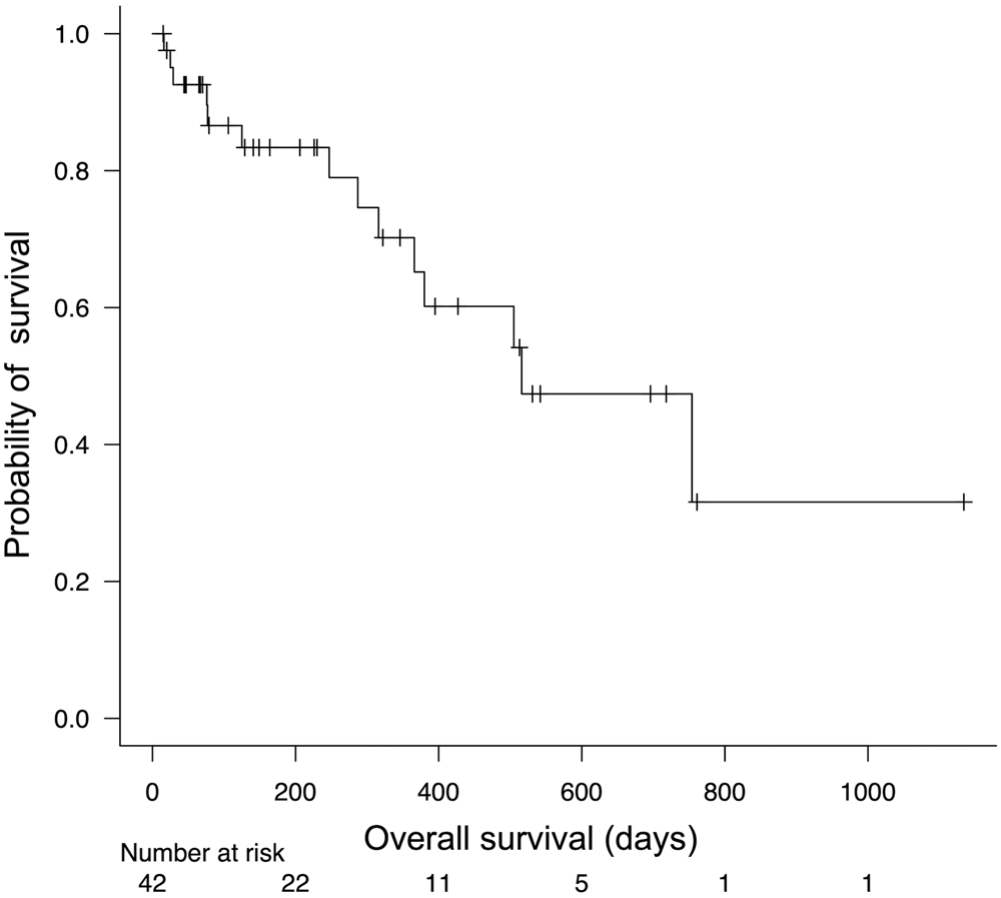
Kaplan–Meier curve for overall patient survival.

The median follow‐up period was 216 (range, 15–1134) days. The Kaplan–Meier curve for overall patient survival is shown in Figure 2. The median survival was 516 days (95% confidence interval [CI], 316 to not applicable days).

### Patient characteristics

The baseline characteristics of the patients who had a median age of 70 (range, 40–90) years are presented in Table 1. The median serum bilirubin level was 3.9 (range, 0.5–21.5) mg/dL. Primary disease included cholangiocarcinoma in 24, gallbladder cancer in four, pancreatic cancer in four, hepatocellular carcinoma in three, metastatic liver cancer in six, and lymph node metastasis in one patient. Six patients exhibited acute cholangitis and jaundice before ERCP was performed. The severity of bile‐duct stricture was classified according to the Bismuth classification. Bismuth I, II, IIIa, IIIb, and IV stenoses were found in six (14.3%), five (11.9%), six (14.3%), three (7.1%), and 22 (52.4%) patients, respectively. Chemotherapy was performed in 20 patients (47.6%) following stent placement. The decision to administer chemotherapy was made by the attending physician based on the patient's performance status and other systemic conditions. Three of the patients had started chemotherapy prior to stent placement. Radiation therapy was performed in one case only. The treatment details according to the disease are presented in Table 2. The chemotherapy regimens and the best therapeutic effects based on the RECIST classification are listed in Table 3.^16^ In the RECIST criteria, 14 patients had partial response, one patient had stable disease, four patients had progressive disease, and one patient had stent occlusion before the assessment of response, which was not possible to determine.

**TABLE 1:** Patient characteristics.

	Patient characteristics
	(*n* = 42)
Age, median (range), years	70 (40–90)
Sex, male/female	28/14
Serum bilirubin (mg/dL)	3.9 (0.5–21.5)
Primary disease, *n* (%)	
Cholangiocarcinoma	25 (59.5)
Gallbladder cancer	5 (11.9)
Pancreatic cancer	4 (9.5)
Hepatocellular carcinoma	3 (7.1)
Metastatic liver cancer	4 (9.5)
Lymph‐node metastasis	1 (2.4)
Presence of acute cholangitis prior to drainage, *n* (%)	6 (14.3)
Bismuth classification, *n* (%)	
I	6 (14.3)
II	5 (11.9)
IIIa	6 (14.3)
IIIb	3 (7.1)
IV	22 (52.4)
Anticancer therapy, *n* (%)	
Chemotherapy	20 (47.6)
Time of chemotherapy initiation	
Before/after inside‐stent placement	3/17

**TABLE 2:** Chemotherapy and radiation therapy.

	Chemotherapy	Radiation therapy
	(Presence/absence, *n*)	(Presence/absence, *n*)
Cholangiocarcinoma, *n* = 24	11/13	1/23
Gallbladder cancer, *n* = 4	4/0	0/4
Pancreatic cancer, *n* = 4	1/3	0/4
Hepatocellular carcinoma, *n* = 3	1/2	0/3
Metastatic liver cancer, *n* = 6	3/3	0/6
Lymph node metastasis, *n* = 1	0/1	0/1

**TABLE 3:** Chemotherapy regimens and therapeutic effects.

		Therapeutic effects
	Chemotherapy regimens	(PR/SD/PD/others, *n*)
Cholangiocarcinoma	Gemcitabine + cisplatin + S‐1	7/0/0/0
	Gemcitabine + cisplatin	1/0/0/0
	Gemcitabine + S‐1	1/0/1/0
	Gemcitabine	0/0/1/0
Gallbladder cancer	Gemcitabine + cisplatin + S‐1	1/0/1/0
	Gemcitabine	1/0/0/0
	Carboplatin + irinotecan	1/0/0/0
Pancreatic cancer	Gemcitabine + nab‐paclitaxel	1/0/0/0
Hepatocellular carcinoma	Atezolizumab + bevacizumab	0/0/1/0
Metastatic liver cancer	Irinotecan + S‐1	1/1/0/0
	Capecitabine + oxaliplatin	0/0/0/1
***Total***		PR: 14; SD: 1; PD: 4; others: 1*

Abbreviations: PD, progressive disease; PR, partial response; SD, stable disease.

^*^One patient had a stent occlusion before the assessment of response.

### Endoscopic procedures and AEs

The median procedure time was 28.5 (11–95) min. Overall, 31 (73.8%) patients underwent unilateral drainage of the right or left liver lobe, and 11 (26.2%) patients underwent bilateral drainage of both the right and left liver lobes. Furthermore, 13 (30.9%) patients underwent ENBD before inside‐stent placement. ENBD placement before inside‐stent placement was performed at the discretion of the surgeon, considering the patient's condition. None of the study patients who underwent ENBD placement had cholangitis. To preserve the sphincter of Oddi function, most patients did not undergo ES; only two (4.8%) patients underwent ES. The endoscopic success rate was 100%, and all patients successfully underwent inside‐stent placement to the target biliary branches. Early AEs were observed in two patients (4.8%; cholangitis, one; pancreatitis, one). Inside‐stent dysfunction was observed in 14 patients (33.3%; obstruction, 11; migration, 3) during the follow‐up periods. Reintervention could be performed with stent replacement via ERCP for 13 of the 14 cases of RBO. In the remaining case, ERCP was attempted but discontinued because of esophageal varices (Table 4).

**TABLE 4:** Procedure and adverse events.

	Procedure and adverse events
	(*n* = 42)
Procedure time, median (range), minutes	28.5 (11–95)
Total success rate (%)	100
***Unilateral/Bilateral stent placement*, *n* (%)**	
Unilateral	31 (73.8)
Left lobe drainage	10 (23.8)
Right lobe drainage	21 (50.0)
Bilateral	11 (26.2)
ENBD management prior to inside‐stent placement, *n* (%)	13 (30.9)
Endoscopic sphincterotomy, *n* (%)	2 (4.8)
Adverse events, *n* (%)	2 (4.8)
Acute cholangitis	1 (2.4)
Acute pancreatitis	1 (2.4)
***RBO*, *n* (%)**	14 (33.3)
Stent obstruction	11 (26.2)
Stent migration	3 (7.1)
***Reintervention for RBO*, *n* (%)**	
Stent replacement via ERCP	13 (31.0)
Conservative therapy	1 (2.3)^a^

Abbreviations: ENBD, endoscopic nasobiliary drainage; ERCP, endoscopic retrograde cholangiopancreatography; RBO, recurrent biliary obstruction.

^a^ A patient in whom ERCP was attempted but abandoned due to esophageal varices.

### TRBO and factors associated with stent dysfunction

**FIGURE 3:**
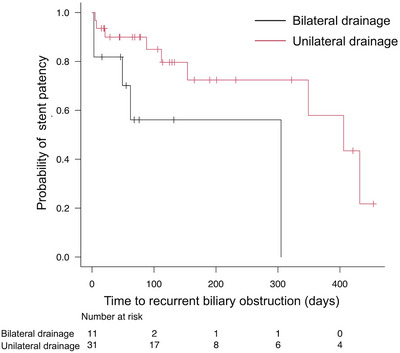
Kaplan–Meier analysis of cumulative stent patency by stent placement.

**FIGURE 4:**
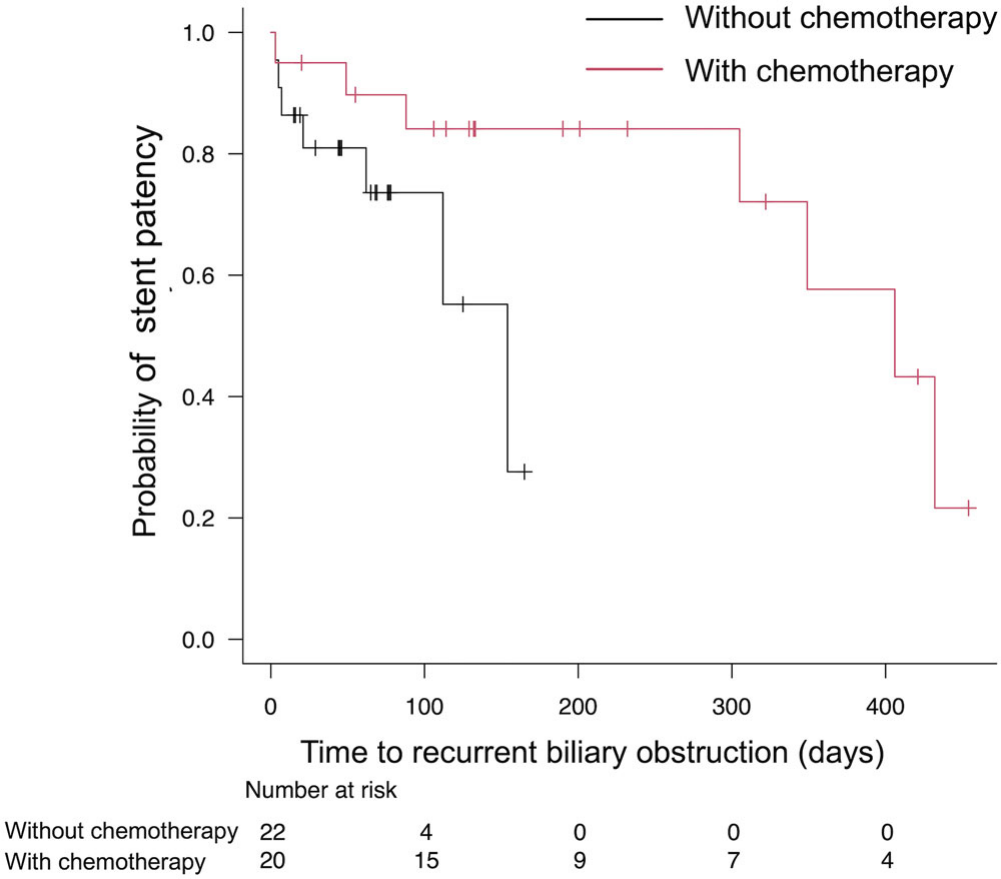
Kaplan–Meier analysis of cumulative stent patency with and without chemotherapy.

Preoperative cholangitis, Bismuth classification (I vs. II, III, and IV), ES, biliary drainage (unilateral or bilateral) method, unilateral drainage lobe (left vs. right), chemotherapy following stent placement, chemotherapy response, ENBD management before inside‐stent therapy, and primary disease (biliary tract cancer vs. nonbiliary tract cancer) were analyzed as factors related to stent patency (Table 5). Univariate analysis (Log‐rank test) revealed significant differences in the stent placement method (406 days in 31 patients with unilateral drainage vs. 305 days in 11 patients with bilateral drainage, *p* = 0.022) and chemotherapy (406 days in 20 patients with chemotherapy vs. 154 days in 22 patients without chemotherapy, *p* = 0.038; Figures 3 and 4). Multivariate analysis (Cox proportional hazards analysis) revealed similar results, and unilateral drainage and chemotherapy were identified as independent factors associated with prolonged stent patency (unilateral drainage [95% CI, 0.07–0.88; hazard ratio {HR} = 0.25; *p* = 0.031] and chemotherapy [95% CI, 0.052−0.99; HR = 0.23; *p* = 0.048; Table 5).

**TABLE 5:** Univariate and multivariate Cox proportional hazard models for stent patency.

		Univariate	Multivariate	HR	95% CI
		***p*‐Value**	***p*‐Value**		
Stent placement method	11, 305 versus 31, 406	0.022	0.031	0.25	0.07–0.88
(bilateral vs. unilateral)					
*n*, TRBO (median), days					
Unilateral drainage lobe	10, 406 versus 21 349	0.866			
(left vs. right)					
*n*, TRBO (median), days					
Chemotherapy	22, 154 versus 20, 406	0.038	0.048	0.23	0.052–0.99
(absence vs. presence)					
*n*, TRBO (median), days					
Chemotherapy effect	5, NA versus 15, 406	0.567			
(PR / SD vs. PD / others)					
*n*, TRBO (median), days					
Bismuth classification	6, NA versus 36, 349	0.392			
(I vs. II, III, and IV)					
*n*, TRBO (median), days					
Endoscopic sphincterotomy	2, 154 versus 40, 349	0.789			
(presence vs. absence)					
*n*, TRBO (median), days					
Acute cholangitis prior to drainage	6, NA versus 36, 349	0.957			
(presence vs. absence)					
*n*, TRBO (median), days					
ENBD management prior to inside‐stent placement	13, 154 versus 29, 406	0.188			
(presence vs. absence)					
*n*, TRBO (median), days					
Primary disease	30, 349 versus 12, 406	0.493			
(biliary vs. nonbiliary tract cancer)					
*n*, TRBO (median), days					

Abbreviations: CI, confidence interval; ENBD, endoscopic nasobiliary drainage; HR, hazard ratio; NA, not applicable; PD, progressive disease; PR, partial response; SD, stable disease; TRBO, time to recurrent biliary obstruction.

## DISCUSSION

This study is the first to report factors affecting stent patency in inside‐stent therapy for patients with MHBO. Interestingly, the results suggest that RBO is less likely to occur with unilateral than with bilateral drainage in the left and right lobes of the liver. Furthermore, the reduction of tumor burden via chemotherapy might contribute to prolonged TRBO. In any case, TRBO longer than that previously reported was obtained for the use of conventional PSs in patients with MHBO. We performed inside‐stent therapy without ES whenever possible, which may have led to better control of the reflux of duodenal juice‐containing bacteria.

Soehendra et al. first reported biliary drainage using PSs placed across the sphincter of Oddi in 1980.^17^ However, some AEs, such as cholangitis due to reflux duodenal juice that contains bacteria, were noted.^18^ Therefore, Pedersen et al. reported “inside”‐stent placement above the sphincter of Oddi.^11^ Subsequently, inside‐stent therapy has been reported to have various advantages, such as preserving the sphincter of Oddi and preventing reflux of duodenal juice and food debris into the bile duct, which enables easy removal and replacement as well as multiple stent placements.^11^, ^19^, ^20^, ^21^

However, stenting methods and stent selection in patients with MHBO remain controversial. The European Society of Gastrointestinal Endoscopy guidelines recommend drainage of ≥50% of the liver volume.^2^ Takahashi et al. reported that >50% drainage is necessary in decompensated cirrhosis, whereas 33% drainage is sufficient in normal livers.^22^ Furthermore, the latest American Society for Gastrointestinal Endoscopy guidelines recommend the drainage of both lobes.^1^ However, in our study, longer TRBO was achieved with simple unilateral drainage than with bilateral drainage. This finding may be because complex bilateral drainage increases the number of intrahepatic bile‐duct branches that can become obstructed. Therefore, it is important to evaluate preoperative images in detail, consider the presence of portal‐vein tumor invasion and liver volume, and develop a strategy to clearly demarcate the drainage areas and sacrificial areas.

Mukai et al. reported that ENBD placement should be performed before stent placement because of the difficulty in assessing liver functional reserve and determining the extent of drainage required. If jaundice resolution and cholangitis suppression are achieved, inside‐stent placement is performed to allow the drainage of the same bile‐duct branches, and if not, an additional stent is added to allow the drainage of another area in addition to the bile‐duct branches drained by ENBD.^23^ As described above, there are no clear guidelines regarding drainage methods for unresectable MHBO. In patients with moderate or severe cholangitis according to the Tokyo guidelines 2018 or severe jaundice, we perform ENBD placement first. However, in patients with no or mild cholangitis, we use an inside stent for initial stent placement. Interestingly, ENBD management before inside stent placement did not affect TRBO. Therefore, performing inside‐stent placement first without ENBD placement may be acceptable in patients with unresectable MHBO.

Various previous reports also have discussed situations wherein a metallic stent or PS is preferable. The Japanese guidelines for biliary tract cancer recommend using uncovered self‐expandable metal stents (UC‐SEMS) or PSs, the European Society of Gastrointestinal Endoscopy guidelines recommend UC‐SEMS and the American Society for Gastrointestinal Endoscopy guidelines recommend UC‐SEMS if the prognosis is <3 months and UC‐SEMS or PSs in other cases.^1^, ^2^, ^3^ The recommended stent also differs among the guidelines. In addition, there have been remarkable advances in chemotherapy for various types of cancer.^4^, ^5^, ^6^ Consequently, the prognosis for the length of survival has increased and reintervention is often performed in the event of stent problems. In fact, some patients with MHBO in this study survived >2 years (Figure 2). Endoscopic reintervention following UC‐SEMS placement is often difficult and may require special treatment, such as percutaneous transhepatic biliary drainage or endoscopic ultrasound‐guided biliary drainage.^1^ However, as mentioned above, an inside stent can be easily removed, and reintervention is relatively easy. For these reasons, we use inside stents initially in patients with MHBO. In fact, in the current study, reintervention via ERCP was achieved in almost all cases of RBO.

Furthermore, in our study, aggressive chemotherapy prolonged TRBO. This result may be because bile‐duct strictures were improved by reducing the tumor burden, thereby reducing the chances of RBO. Interestingly, even in cases wherein tumor shrinkage was achieved, stent migration was infrequent (Table 4). This finding may be because of the stent shape, which is designed to follow the bile‐duct shape. These stents are available with strong or weak bending angles and can be used depending on the bile‐duct shape (Figure 1). In our study, the therapeutic effect of chemotherapy did not affect TRBO. This result contradicts the finding that chemotherapy prolongs TRBO. However, considering the limited number of cases with only four PD cases, finding a statistical association between the therapeutic effect of chemotherapy and TRBO may not be possible. In addition, although we evaluated the therapeutic effect of chemotherapy as accurately as possible according to the RECIST criteria, MHBO is a complex condition, and discrepancies between the therapeutic effect and the rate of improvement in bile duct stenosis may exist.

This study had several limitations. First, this was a single‐center retrospective study, and the number of cases analyzed was relatively small. Thus, selection bias may have occurred in the choices of drainage method, number of stents, and chemotherapy. In particular, the number of stents and the choice of drainage area were precisely determined based on imaging studies, but the endoscopist's discretion may have greatly influenced the outcome. It is desirable to conduct prospective studies with a larger number of cases in the future to overcome these biases. Second, we excluded patients with poor improvement of jaundice and analyzed only those in whom jaundice was successfully resolved. One of the two patients who were excluded due to poor improvement in jaundice had initially undergone unilateral drainage but later switched to bilateral drainage. Another patient exhibited multiple liver metastases from colorectal cancer, and their condition led to a suspicion of liver failure. Third, this study included eight patients in whom the stent was replaced before RBO occurred. In recent years, multidrug chemotherapy has emerged for biliary tract cancer, resulting in a strong immunosuppressive state in patients. Therefore, RBO occurrence during chemotherapy often leads to severe cholangitis. Later in the study period, an increasing number of patients underwent periodic stent replacement every 3–4 months to avoid chemotherapy interruption caused by severe cholangitis. The TRBO duration was longer than that reported in previous reports,^24^, ^25^ possibly partly because of the inclusion of patients who received periodic stent replacement. Fourth, we were not able to measure liver volumes using dedicated software. Thus, we may have cases in which less than 50% of the liver area was drained, especially in those with unilateral drainage. Nevertheless, the clinical success rate of initial inside‐stent placement in our study was 95.5%. The accurate assessment of blood flow in the portal vein and hepatic arteries during preoperative imaging and the clear distinction between areas that required drainage and those that did not may have contributed to efficient drainage. Areas of the liver that have already lost portal blood flow may not have needed to be drained considering that little liver function is thought to remain, even if the volume is large.

In conclusion, in patients with unresectable MHBO, simple unilateral drainage, and early aggressive chemotherapy may prolong TRBO.

## CONFLICT OF INTEREST STATEMENT

None.

## ETHICS STATEMENT

**Approval of the research protocol by an Institutional Reviewer Board**: This study was approved by the Institutional Review Board of Nara Medical University Hospital (IRB No: 2701) and performed according to the Helsinki Declaration of the World Medical Association.

## INFORMED CONSENT

Written informed consent was obtained from all patients before performing ERCP. An opt‐out option on the website was used instead of written informed consent for inclusion in this study owing to the retrospective nature of this study.
